# Activation of acid‐sensing ion channels by carbon dioxide regulates amygdala synaptic protein degradation in memory reconsolidation

**DOI:** 10.1186/s13041-021-00786-7

**Published:** 2021-05-07

**Authors:** Boren Lin, Khaled Alganem, Sinead M. O’Donovan, Zhen Jin, FarzanehSadat Naghavi, Olivia A. Miller, Tyler C. Ortyl, Ye Chun Ruan, Robert E. McCullumsmith, Jianyang Du

**Affiliations:** 1 Department of Anatomy and Neurobiology, The University of Tennessee Health Science Center, 38163 Memphis, TN USA; 2 Department of Biological Sciences, The University of Toledo, 43606 Toledo, OH USA; 3 Department of Neurosciences, The University of Toledo Medical Center, 43614 Toledo, OH USA; 4 Department of Biomedical Engineering, Faculty of Engineering, The Hong Kong Polytechnic University, Hong Kong, People’s Republic of China; 5 Neurosciences Institute, ProMedica, OH 43614 Toledo, USA; 6 Neuroscience Institute, The University of Tennessee Health Science Center, 38163 Memphis, TN USA

**Keywords:** Carbon dioxide, Acid‐sensing ion channels, Reconsolidation, Aversive conditioning, Memory retrieval, AMPA receptors, Ubiquitination, Proteasome, Protein degradation

## Abstract

Reconsolidation has been considered a process in which a consolidated memory is turned into a labile stage. Within the reconsolidation window, the labile memory can be either erased or strengthened. Manipulating acid-sensing ion channels (ASICs) in the amygdala via carbon dioxide (CO_2_) inhalation enhances memory retrieval and its lability within the reconsolidation window. Moreover, pairing CO_2_ inhalation with retrieval bears the reactivation of the memory trace and enhances the synaptic exchange of the calcium-impermeable AMPA receptors to calcium-permeable AMPA receptors. Our patch-clamp data suggest that the exchange of the AMPA receptors depends on the ubiquitin-proteasome system (UPS), via protein degradation. Ziram (50 µM), a ubiquitination inhibitor, reduces the turnover of the AMPA receptors. CO_2_ inhalation with retrieval boosts the ubiquitination without altering the proteasome activity. Several calcium-dependent kinases potentially involved in the CO_2_-inhalation regulated memory liability were identified using the Kinome assay. These results suggest that the UPS plays a key role in regulating the turnover of AMPA receptors during CO_2_ inhalation.

## Introduction

Being able to predict threats by recollecting fear stimuli is crucial for most animals such that one can behave adaptively to the dangerous environmental situation [1]. However, since fear memories developed due to traumatic events may trigger mental health conditions, attenuating such memories could be a solution to its resulting psychiatric disorders [2]. Pavlovian conditioning is a common method to develop a reflex response by training with repetitive actions. It was used to assess the ability of lab mice to learn and remember an association between a conditioned stimulus (CS, i.e. an auditory cue) and an aversive unconditioned stimulus (US, i.e. an electric foot shock), and how that memory can be modified [3]. A retrieval cue (CS alone) can recall and destabilize the previously coupled US-induced aversive memory, and at this labile state, the memory is subjected to diminish by the following extinction process, in which the animal receives consecutive auditory cues without coupling with foot shocks [4, 5].

Our earlier studies demonstrated that transient acidification by exposing animals to CO_2_ in conjunction with the retrieval cue significantly enhanced labilization of the target memory and more effectively weakened the aversive memory after memory extinction [6]. This regulatory effect was mediated by acid-sensing ion channels (ASICs), non-voltage gated Na^+^- and Ca^2+^-permeable channels that can be activated by extracellular acidosis [6]. During retrieval, increased rectification suggested that AMPA receptors (AMPARs) switched from Ca^2+^-impermeable (CI) to Ca^2+^-permeable (CP) to promote neuroplasticity, and CO_2_ inhalation during retrieval augmented the exchange of AMPA receptors and further energized Ca^2+^ influx [7]. Our data suggested that inhaling CO_2_ while the retrieval tone was presented caused more rectification than retrieval alone. In contrast, adding CO_2_ to retrieval in ASIC1a^−/−^ mice failed to increase rectification [6].

However, the molecular signaling pathway that involves the CO_2_-induced lability of the retrieved memory is still not clear. Recent studies in the reconsolidation window suggested that the UPS, which controls the majority of protein turnover in mammals, is required for synaptic protein degradation, synaptic plasticity, and long-term memory [8]. Upon retrieval in the reconsolidation window, the consolidated memory has been considered destabilized and requires new protein synthesis to restabilize the labile memory [4, 9]. Many intracellular Ca^2+^-dependent and -independent signaling pathways ( e.g. mTOR, MAPK and PKA) mediated protein translation involve in reconsolidation and inhibition of these pathways results in alternation in long-term memory within the original memory trace [10–13]. Also, Ca^2+^ and its binding proteins have been shown required for the activation of the UPS [14].

Combined the backgrounds with our previous studies, we hypothesized that CO_2_ inhalation in memory retrieval enhances UPS, particularly in targeting AMPAR subunit GluR2 to increase the exchange of Ca^2+^ impermeable-AMPARs (CI-AMPARs) to Ca^2+^-permeable-AMPAR (CP-AMPARs). The outcome of this work might update our current understanding of the mechanism of memory reconsolidation.

## Materials and methods

### Mice

Both male and female 9–12 weeks old C57BL6 mice and ASIC1a^−/−^ mice were randomly selected for the experiment groups. The C57BL6 mice were ordered from the Jackson Laboratory and maintained in our animal facilities. The ASIC1a^−/−^ mice were gifted from Drs. Michael Welsh and John Wemmie’s laboratories at the University of Iowa and maintained in our animal facilities. Experimental mice were maintained on a standard 12-hour light-dark cycle and received standard chow and water ad libitum. Animal care and procedures met the National Institutes of Health standards. The University of Tennessee Health Science Center Laboratory Animal Care Unit (Protocol #19-0112) and University of Toledo Institutional Animal Care and Use Committee (Protocol #108791) approved all procedures.

### Auditory aversive conditioning and retrieval

All mice underwent habituation by handling for 15 min each day, 3 days before aversive conditioning. On day 1, mice were habituated to the aversive conditioning chamber for 7 min (Med Associates Inc.). Then, the mice were presented with six pure tones (80 dB, 2 kHz, 20 s each) paired with 6-foot shocks (0.7 mA, 2 s, 100 s interval) in context A. After aversive conditioning, the mice were returned to their home cage. On day 2, 50 µM Ziram was injected into the amygdala of the mice (see details in surgery and chemical infusion next section). The mice were then returned to their home cage until recovery. One hour later, the mice were placed into a new environment (context B) and inhaled either air or 10 % CO_2_ (10 % CO_2_ + 21 % oxygen, balanced with nitrogen) for 7 min. Five minutes after inhalation of air or 10 % CO_2_, a 20-second pure tone was used to retrieve the aversive memory. The mice were then returned to their home cages. Ten minutes later, the mice were euthanized, and brain slices were dissected for patch-clamp and western-blot experiments. To evaluate the outcomes of freezing behavior, the percentage of freezing time during the pure tone presentation was scored automatically using VideoFreeze software (Med Associates Inc.) [6].

## Surgery and chemical infusion

For the Ziram injection procedure, mice were anesthetized using an isoflurane anesthetic vaporizer, secured to a Model 942 stereotaxic instrument (KOPF instruments). 1 µL Ziram (50 µM, diluted in saline) was injected bilaterally into the amygdala (relative to bregma: − 1.2 mm anterioposterior; ±3.5 mm mediolateral; − 4.3 mm dorsoventral) using a 10 µl Hamilton microsyringe and a WPI micro-4 microsyringe pump [6, 15]. Mice were then recovered for 1 h followed by the retrieval procedure and then the brain slices were dissected for patch-clamp recordings and western-blot experiments. For the Clasto-Lactacystin β-lactone (β-Lac, Cayman Chemical, Ann Arbor, MI) injection, mice were anesthetized with isoflurane through an anesthetic vaporizer, secured to a Model 942 stereotaxic instrument (KOPF instruments) and a cannula made from a 25-gauge needle was inserted bilaterally into lateral amygdala (relative to bregma: − 1.2 mm anterioposterior; ± 3.5 mm mediolateral; − 4.3 mm dorsoventral) [6, 15]. Dental cement secured the cannula and bone anchor screw in place. Mice recovered for 4–5 days before any subsequent testing was carried out. A 1 µL Hamilton syringe connected to a 30-gauge injector was inserted 1 mm past the cannula tip to inject 0.3 µL β-Lac at 32ng/µl (dissolved in DMSO in1 µl and further diluted in PBS) bilaterally [16, 17]. The injection sites were mapped post-mortem by sectioning the brain (10 μm coronal) and performing cresyl violet staining.

### Brain slice preparation and patch‐clamp recording of amygdala neurons

Ten minutes after the memory retrieval procedure, mice were euthanized with overdosed isoflurane, and the whole brains were dissected into pre-oxygenated (5 % CO_2_ and 95 % O_2_) ice-cold high sucrose dissection solution containing (in mM): 205 sucrose, 5 KCl, 1.25 NaH_2_PO_4_, 5 MgSO_4_, 26 NaHCO_3_, 1 CaCl_2_, and 25 glucose [6]. 300 μm sections were dissected using a vibratome (Leica VT-1000 S) and incubated in ACSF containing (in mM): 115 NaCl, 2.5 KCl, 2 CaCl_2_, 1 MgCl_2_, 1.25 NaH_2_PO_4_, 11 glucose, 25 NaHCO_3_ bubbled with 95 % O_2_/5 % CO_2_, pH 7.35 at room temperature (20-22 °C) at least 1 h before patch-clamp recordings. Slices were continuously perfused with 5 % CO_2_ / 95 % O_2_ ACSF (~ 3.0 ml/min) in a recording chamber throughout the experiments [6].

Pyramidal neurons in the lateral amygdala were selected under an Olympus BX51WI upright microscope. The pipette solution for whole-cell patch-clamp recordings contains (in mM): 135 Cs-SO_3_CH_3_, 5 NaCl, 10 HEPES, 4 MgATP, 0.3 Na_3_GTP, 0.5 K-EGTA (mOsm = 290, adjusted to pH 7.25 with CsOH). The pipette resistance (measured in the bath solution) was 4–6 MΩ. High-resistance (> 1 GΩ) seals were formed in voltage-clamp mode. Picrotoxin (100 µM) was added to the ACSF throughout the recordings to avoid inhibitory responses. To record the AMPAR rectification and 1-naphthylacetyl spermine (NASPAM) sensitive currents,100 µM D-2-amino-5-phosphonovalerate (D-APV) was added to block the NMDAR-conducted excitatory postsynaptic currents (EPSCs). The EPSCs were measured ranging from − 80 to + 60 mV with a 20 mV step. The ratio of the amplitude of EPSCs at − 80  and + 60 mV was measured to determine the rectification index. 50 µM NASPM was added to the bath solution to detect the NASPAM sensitive AMPAR-EPSCs, voltage was holding at − 80mV. EPSCs were acquired at 10 kHz using Multiclamp 700B and pClamp 10.1. EPSCs were analyzed using Clampfit 10.1 [6].

### Immunoprecipitation (IP) and Western-blot analyses

Tissue lysates were prepared by homogenization using pellet pestles in cold IP lysis buffer composed of 25 mM Tris-HCl pH 7.4, 150 mM NaCl, 1 mM EDTA, 1 % NP-40 and 5 % glycerol with protease and phosphatase inhibitors (Thermo Scientific, Waltham, MA) followed by centrifugation at 12,000 rpm for 15 min at 4 °C to remove debris. GluR2 in the whole cell lysate was concentrated by IP using monoclonal anti-GluR2 antibody and protein A/G agarose, both obtained from Thermo Scientific. To prepare synaptic protein extracts, tissue samples were homogenized in Syn-PER in synaptic protein extraction reagent (Thermo Scientific, Waltham, MA) on ice followed by serial centrifugations per manufacturer’s instructions to isolate synaptosomes.

For Western-blot detection, samples were normalized for equal protein loading, boiled in sodium dodecyl sulfate (SDS) Laemmli sample buffer, resolved by SDS-polyacrylamide gel electrophoresis, transferred to polyvinylidene difluoride membranes and immunoblotted with antibodies specific for the proteins of interest. Polyclonal anti-phospho-Rpt6 antibody was obtained from Signalway, College Park, MD, and polyclonal anti-CaMKII and anti-Actin antibody and mouse control IgG from Cell Signaling, Danvers, MA.

### 20s proteasome activity assay

Activities of the catalytic core of the proteasome complex 20s were measured by a 20s proteasome activity kit (MilliporeSigma, Burlington, MA) utilizing a fluorophore-labeled 20s substrate LLVY-AMC. Tissue lysates were prepared by homogenization using pellet pestles in cold T-PER tissue protein extraction reagent (Thermo Scientific, Waltham, MA) followed by centrifugation at 12,000 rpm for 15 min at 4 °C to remove debris. The assay was performed according to the manufacturer’s instructions in a 96-well fluorometer plate. Some samples collected from animals without behavior treatment were pre-incubated with a proteasome inhibitor Lactacystin for 15 min at room temperature before adding 20s substrate. After a 2 h incubation at 37 °C, fluorescence was measured at 380/460 nm.

### Kinome assay

All materials and software were obtained from PamGene, ‘s-Hertogenbosch, Netherlands. The kinase activity profiling platform, PamStation12, was used to measure serine/threonine kinome activity using the STK PamChip. Each array on the STK PamChip contains 141 serine/threonine reporter peptides that are immobilized on a porous membrane. The assay was performed based on the PamStation12 standard protocols. Briefly, Pooled samples were diluted to 0.2 µg/µL and ran in duplicates on the kinome array, using 2 STK chips. Identical protein amounts were loaded for each condition. It was performed by blocking each well with 2 % bovine serum albumin (BSA) before 2 µg of protein of the samples, and 157 µM adenosine triphosphate (ATP), and a primary antibody mixture as a part of the two-step reaction process specifically designed for STK PamChips. FITC-labeled anti-phospho serine-threonine antibodies were added to each well. The homogenized samples alongside the assay mix were pumped through the wells through timed cycles to speed up the phosphorylation reaction between kinases in the samples and the reporter peptides on the chip. The degree of phosphorylation for each peptide in each well was measured in real-time using Evolve kinetic image capture software. The Evolve software captures images of FITC-labeled anti-phospho antibodies binding to each phosphorylated peptide substrates. Peptide spot intensities were captured across multiple exposure times (10, 20, 50, 100, 200 ms) during the post-wash phase. The BioNavigator software was used to convert the captured images to numerical values based on the intensity levels to be used for the kinome analysis.

The Kinome Random Sampling Analyzer (KRSA) software tool was used to process, analyze, and visualize the kinome data [18]. KRSA was used to calculate the linear regression slope (signal to exposure time) and scale the values by multiplying them by 100. The logged transformed values were used as the final signal (i.e. peptide phosphorylation intensity) in comparative analyses. Quality control steps were used to remove peptides with either a very low signal (signal < 5) or R-squared of less than 0.9 of the linear models. The signal ratio between pairs of group conditions was used to calculate log2 fold change (LFC) for each peptide. The LFC was calculated per chip and then averaged across the chips. Peptides with an LFC of at least 0.2 were carried forward to the upstream kinase analysis. For the upstream kinase analysis, KRSA takes these lists of peptides and uses a random resampling approach to identify implicated kinases using multiple kinase-substrate databases [18].

### Statistical analysis

Nonparametric unpaired Mann–Whitney test was used to compare the mean results of two groups. One-way ANOVA and Tukey’s post-hoc multiple comparison tests were used for comparing results more than two groups. p < 0.05 was considered statistically significant and labeled as an asterisk in the figures. Graphpad Prism 8 software was used to analyze statistical data. Data were presented as means ± SEM. Sample sizes (n) are indicated in the figure legends, and data are reported as biological replicates (data from different mice, different brain slices). Each group contained tissues pooled from 3 to 5 mice.

## Results

### **Inhibition of ubiquitination prevents CO**_**2**_**and retrieval-induced AMPAR-EPSC rectification**

**Fig. 1:**
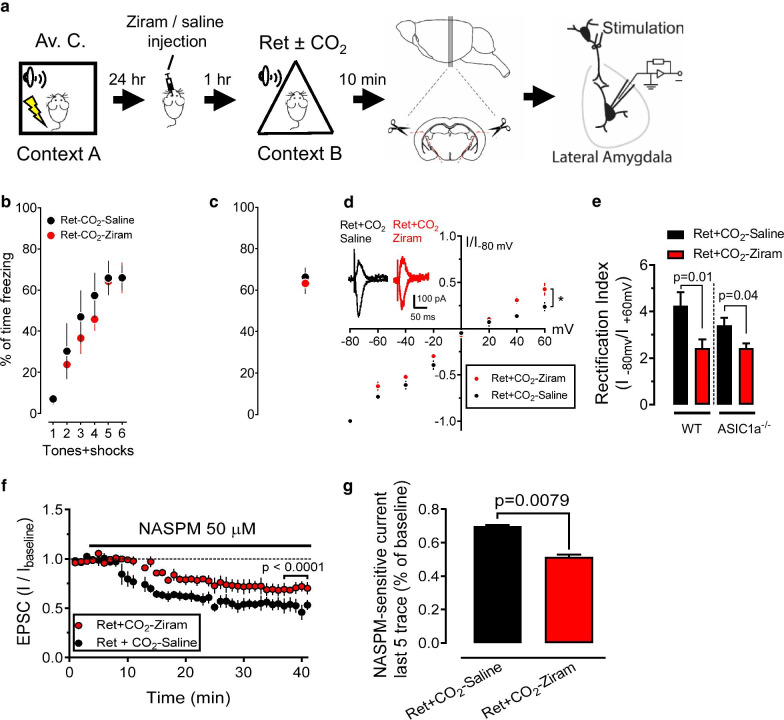
Inhibition of ubiquitination prevents CO_2_ and retrieval-induced AMPAR- EPSC rectification.** a** Experimental procedure of aversive conditioning (Av. C.), memory retrieval (Ret), amygdala slices collection, and patch-clamp recording. On day 1, the mice were subjected to 6 pure tones, paired with 6 foot shocks in context A. One day after, 50µM Ziram or saline was injected into the amygdala bilaterally and then returned to their home cages. One hour later, the mice were placed in context B and subjected to one pure tone as retrieval. 10 min after retrieval, the mice were euthanized, and the amygdala slices were collected and incubated in ACSF for patch-clamp recordings. **b** Percentage of freezing time during the CSs in aversive conditioning. **c **Percentage of freezing time during the CSs in memory retrieval. **d** AMPAR-EPSC current-voltage relationship in recorded neurons in saline and Ziram injection groups. Insets show an example of the AMPAR-EPSCs in − 80 mV and + 60 mV in each group. **e** Effects of Ziram on the AMPAR-EPSC rectification index (current at − 80 mV / current at + 60 mV) in the WT and the ASIC1a^−/−^ mice groups. **f** Application of 50 µM NASPM on the AMPAR-EPSCs. D-APV (100 µM) was added to the bath solution throughout the experiments to block NMDA currents and Picrotoxin (100 µM) was added to the ACSF throughout the recordings to avoid inhibitory responses. **g** Relative NASPM-sensitive currents, average of the last 5 recordings in each group (% of baseline) were compared in the Ziram and saline injection groups. Data are mean ± SEM. n = 8–10 cells in each group. p values are indicated in each panel, by nonparametric unpaired Mann–Whitney test

Protein degradation and synthesis are critically involved in the process of reconsolidation [4, 9]. Memory retrieval results in time-dependent endocytosis of AMPAR subunits GluR1 and GluR2, which is observed within 6 h after retrieval [7, 19, 20]. Our previous data suggested that CO_2_ inhalation in memory retrieval enhances the lability of the memory and boosts the efficiency of the memory erasure through enhancing the exchange from CI-AMPA receptors to CP-AMPARs [6]. However, little is known about the molecular mechanism involved in the CO_2_ inhalation induced AMPARs turnover. Recent evidence suggests that the increased UPS activity regulates protein degradation and is critical for regulating memory reconsolidation [16, 21]. To further test if the UPS involves in the CO_2_ inhalation-induced protein turnover, we measured the rectification of AMPARs (the signature of the CP-AMPARs) after CO_2_ inhalation and retrieval with Ziram, a ubiquitin E1 ligase inhibitor [22]. Twenty-four hours after aversive conditioning, Ziram (50µM) or saline was injected into the amygdala bilaterally 1 h before the memory retrieval (Fig. 1a). The data of aversive conditioning and retrieval were shown in Fig. 1b, c. In the saline injection group, we found that 10 % CO_2_ inhalation paired with retrieval induces a strong current rectification of AMPARs, indicating a sufficient number of CP-AMPARs was produced [6]. Whereas, the rectification was reduced in the Ziram injection group (Fig. 1d). This data indicated that the production of CP-AMPARs is ubiquitination dependent. In addition, we found that Ziram inhibited less AMPAR-EPSC rectification in the ASIC1a^−/−^ neurons than that in the WT group, suggesting that the CO_2_-induced AMPAR turnover is ASIC1a dependent (Fig. 1e). To further confirm the above result, we applied a specific CP-AMPARs inhibitor, 1-naphthylacetyl spermine (NASPM), to test the CP-AMPAR EPSCs after memory retrieval and CO_2_ inhalation. Compared to the saline injection group, 50 µM ziram decreased NASPM-sensitive EPSCs (Fig. 1f, g). This data supports the conclusion that CO_2_ inhalation and retrieval-induced CP-AMPARs are ubiquitination dependent.

### **CO**_**2**_**inhalation in aversive memory retrieval potentiates ubiquitination**

**Fig. 2:**
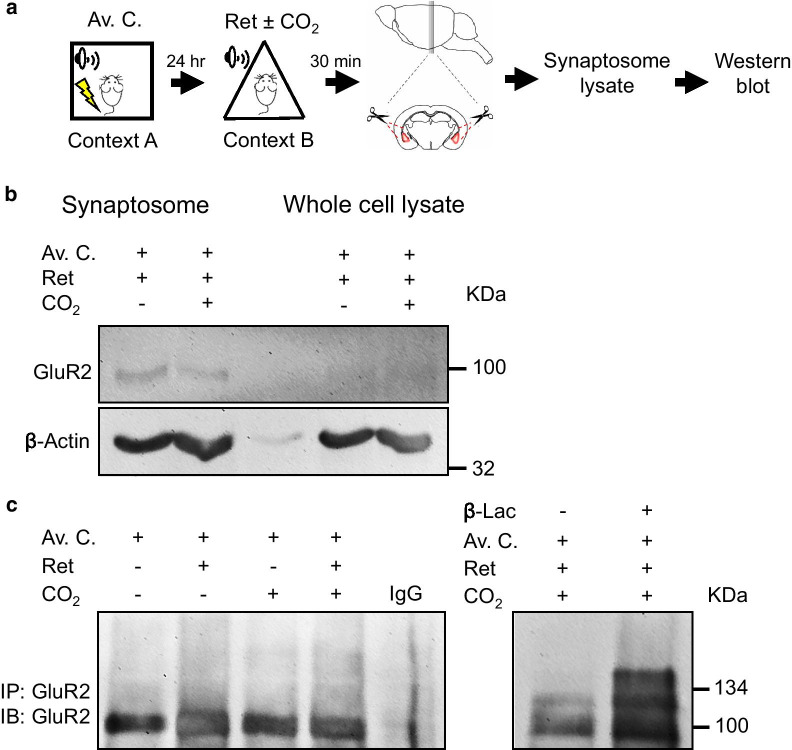
CO_2_ inhalation during retrieval promotes GluR2 ubiquitination and degradation. **a** Schematic of experimental procedure of aversive conditioning (Av. C.), memory retrieval (Ret) and CO_2_ inhalation, as described previously. Thirty minutes after, brain slices were prepared using a brain matrix, and amygdala isolated by a biopsy punch. Synaptosomal proteins were collected by using Syn-PER Reagent. Whole tissue lysates or synaptosomal proteins were subjected to SDS-PAGE and analyzed by Western blot. **b** Synaptosomal proteins collected from animals receiving aversive conditioning with or without Ret and with or without CO_2_ are indicated. GluR2 or actin was detected by using anti-GluR2 or anti-actin antibody respectively. **c** GluR2 in whole tissue lysates collected from animals subjected to aversive conditioning and Ret/CO_2_ was first concentrated by IP using protein A/G agarose in conjunction with monoclonal anti-GluR2 antibody. Ubiquitinated GluR2 was assessed by Western blot analysis. Some animals were given β-Lac after behavior treatment. The negative control was prepared using nonspecific mouse IgG. Data are representative of three independent experiments

Switching CI-AMPARs to more excitatory CP-AMPARs via ubiquitination, endocytosis and proteasomal degradation of AMPAR subunit GluR2 at glutamatergic synapses is essential for neuroplasticity [7, 20]. By introducing a CI-AMPAR endocytosis inhibitor injected into the mouse lateral amygdala before memory retrieval, we have demonstrated that the effect of CO_2_ on conditioned freezing in the spontaneous recovery and renewal memory tests was attenuated. This data implicated that the endocytosis of AMPARs driving the exchange of its Ca^2+^-impermeable to preamble form may be enhanced by CO_2_-induced acidification and ASICs [6]. Since the presence of subunit GluR2 determines AMPAR’s Ca^2+^ permeability, relevant molecular events on GluR2 were investigated in this study. Amygdala from mice receiving aversive conditioning, retrieval, and/or 10 % CO_2_ inhalation or sham controls were harvested, whole-cell and synaptosome lysates were prepared and Western blot analysis was conducted (Fig. 2a). The level of GluR2 in the synaptosome suggested a decrease of this AMPAR subunit in response to CO_2_ inhalation (Fig. 2b). The ubiquitinated GluR2 was found more robust in mice exposed to CO_2_ during retrieval when a proteasome inhibitor was used to block the protein degradation (Fig. 2c).

**Fig. 3:**
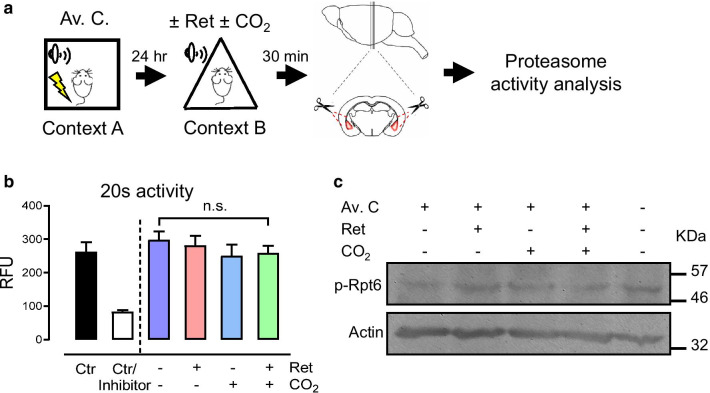
CO_2_ inhalation does not directly affect proteasome activity.** a** Schematic of experimental procedure of aversive conditioning (Av. C.), memory retrieval (Ret) and CO_2_ inhalation, as described previously. Thirty minutes later, brain slices were prepared using a brain matrix, and amygdala isolated by a biopsy punch. Whole tissue lysates were subjected to proteasome activity analysis. **b** Samples collected from animals receiving aversive conditioning with or without Ret and with or without CO_2_ are indicated. Proteasome activity in cell lysates was measured by a fluorometric proteasome 20s assay kit. Samples prepared from animals without behavioral treatment served as controls, and a proteasome inhibitor was used to confirm the specificity. **c** Tissue samples were collected from animals subjected to aversive conditioning and Ret/CO_2_. Rpt6 phosphorylation was assessed by SDS-PAGE and Western Blot analysis using anti-phospho-Rpt6 and anti-actin antibodies. Values are expressed in mean ± SEM of three independent experiments. ‘n.s.’ indicates not statistically significant by one-way ANOVA with Tukey’s post-hoc multiple comparisons

Surprisingly, CO_2_ inhalation did not affect proteasome activity measured by 20s core particle activity assays and Western blot analysis for phosphorylation of the 19s regulatory particle Rpt6 (Fig. 3). Although recent studies suggested that the proteasome activity was increased following aversive memory acquisition [16, 21], our data did not support that the increase of proteasome activity is required for either memory retrieval or CO_2_ inhalation. These results indicated that when the retrieval-induced lability of an aversive memory was enhanced by CO_2_ inhalation, ubiquitin-mediated protein degradation on AMPAR subunit GluR2 was increased without changing the proteasome activity, leading to an increase of Ca^2+^ permeability of AMPARs.

### **Potential Ca**^**2+**^**-dependent protein kinases that might be involved in the CO**_**2**_**inhalation and retrieval-induced protein degradation**

**Fig. 4:**
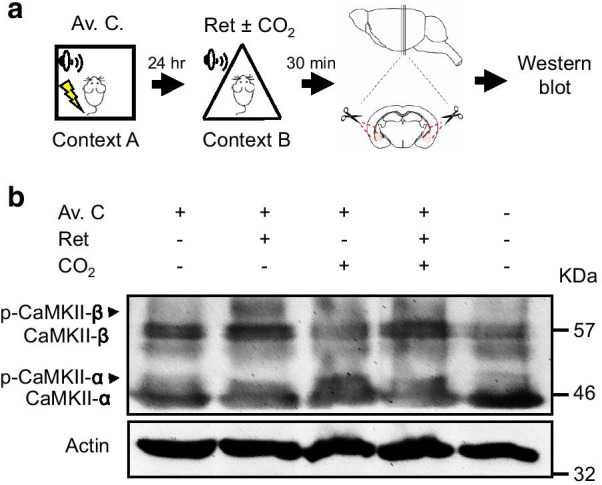
CO_2_ inhalation during retrieval does not alter CaMKII level. **a** Schematic of experimental procedure of aversive conditioning (Av. C.), memory retrieval (Ret) and CO_2_ inhalation, as described previously. Amygdala samples were collected for Western-blot analysis after retrieval. **b** Protein levels of CaMKII-α, CaMKII-β, phosphorylated (p) CaMKII-α, p-CaMKII-β and actin were assessed by Western-blot analysis using anti-CaMKII and anti-actin antibodies. Data are representative of three independent experiments

**Fig. 5:**
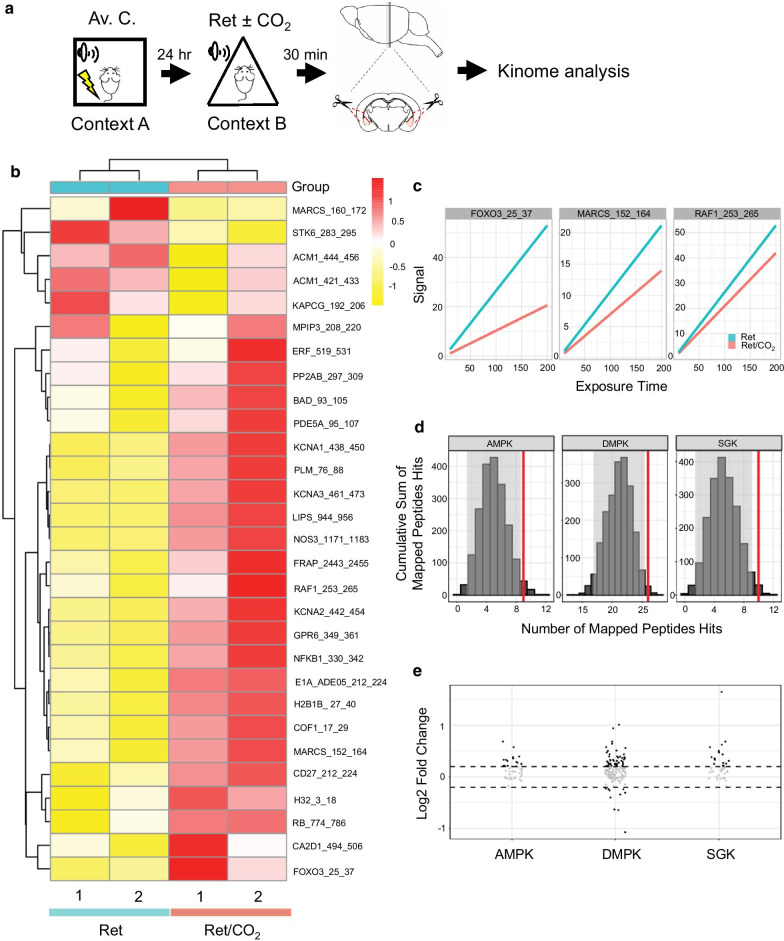
Assessment of CO_2_ inhalation and retrieval-activated Ca^2+^-dependent protein kinases using Kinome assay. **a** Schematic of experimental procedure of aversive conditioning (Av. C.), memory retrieval (Ret) and CO_2_ inhalation, as described previously. Ten minutes later, brain slices were prepared using a brain matrix, and amygdala isolated by a biopsy punch. Whole tissues were subjected to kinome analysis. **b** A heatmap with hierarchical unsupervised clustering representing relative signal intensities of differentially phosphorylated peptides between memory retrieval and retrieval plus CO_2_ samples. Peptides were selected using 0.2 as the log2 fold change cutoff. **c** The linear model fit of the signal intensity as a function of the exposure time for three selected peptides. **d** The random sampling analysis output showing the expected distribution of mapped peptides to kinases based on a random selection of peptides (histogram) and the standard deviation (gray box represents two standard deviations from the mean) versus the observed number of peptides hits (red line). Red lines outside of the distribution indicate significant hits. **e** Log2 fold change values of peptides signals. Peptides that are mapped to AMPK, DMPK, and SGK show increased activities in the retrieval plus CO_2_ group

Although intracellular Ca^2+^ is important for the CO_2_ inhalation enhanced memory lability and exchange of AMPARs [6], surprisingly, the activation of CaMKII, one of the kinases known to mediate signal cascades in learning and memory [23], was not further augmented (Fig. 4). To clarify how Ca^2+^ involves in the signaling pathways, we explored other Ca^2+^-dependent proteins. Since protein kinases are essential signaling components involved in various neuronal functions, we then investigated the potential impact on kinase activities due to transient acidification, amygdala tissues from mice subjected to aversive conditioning followed by memory retrieval with or without CO_2_ inhalation were harvested, lysed, and subjected to kinome analysis by using an array representing 141 reporter peptides as substrates for a wide spectrum of serine/threonine protein kinases (Fig. 5a) [18, 24]. Phosphorylation on each peptide was detected in real-time using fluorescently labeled antibodies at different exposure times. The fold change in fluorescence intensity of each peptide between two sample groups was computed. Peptides that were undetectable, exhibited a non-linear signal-time relationship or showed an LFC lower than 0.2 were excluded. From these phosphorylated peptides, their responsible upstream kinases were identified, and among them, kinases with observed hits greater than two standard deviations from the distribution means (Z-Score > 2), derived from 2000 iterations of random sampling analysis, were recognized as more active (retrieval vs. retrieval plus CO_2_) (Fig. 5b). SGK, DMPK and AMPK are Ca^+^-regulated kinases and were found more active in amygdala samples obtained from animals receiving CO_2_ inhalation (Fig. 5c–e), further supporting the role of ASICs for memory lability, and suggesting possible mediators of this process.

## Discussion

**Fig. 6:**
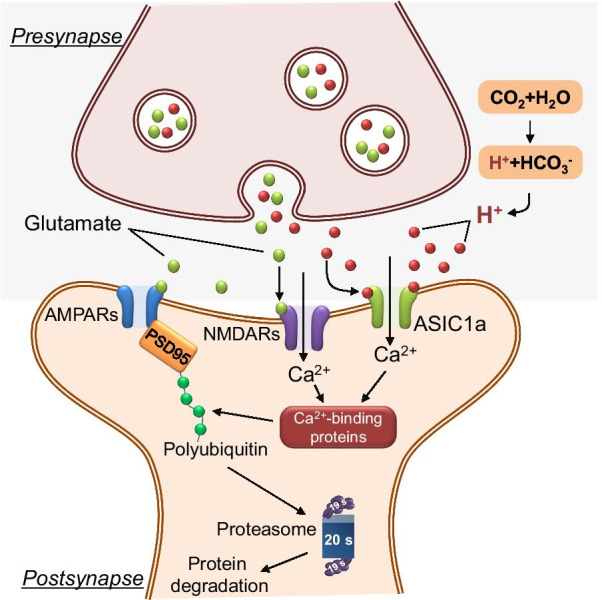
A hypothesized model of how CO_2_ inhalation and ASICs regulate protein degradation after memory retrieval. CO_2_ inhalation decreases the brain pH during memory retrieval. The low pH activates ASICs on the postsynaptic membrane, results in the increase of intracellular Ca^2+^. Then Ca^2+^ can bind to its binding proteins and facilitate the ubiquitination process and eventually the polyubiquitinated GluR2 was degraded by the proteasome or other enzymes. The lack of GluR2 in the new synthesized AMPARs indicated the outcomes of the exchange of CI-AMPARs to CP-AMPARs

In summary, our data suggest that (1) the CO_2_ inhalation-enhanced AMPAR turnover in reconsolidation window is UPS dependent; (2) CO_2_ inhalation enhances ubiquitin-mediated GluR2 degradation through the UPS without affecting the proteasome activity; (3) Although CaMKII is not involved in the CO_2_ inhalation-enhanced protein degradation, several Ca^2+^-dependent kinases might be involved in the process. These results provide evidence to support the hypothesis that the application of CO_2_ inhalation, through activating ASICs, within the reconsolidation window enhances AMPAR turnover through promoting the intracellular Ca^2+^-dependent UPS (Fig. 6). Of course, we can not conclude that the effects of CO_2_ on protein degradation are entirely ASIC-dependent since the functions of CO_2_ inhalation in the brain are extensive. More works are necessary to identify the specific effects of CO_2_ on the reconsolidation process.

Numerous studies supported the hypothesis that a stabilized memory returns into a labile stage after memory retrieval called memory reconsolidation [4, 19, 25]. Protein degradation and new protein synthesis are involved in this process [4, 26]. Recent evidence suggests that the UPS-dependent protein degradation is required for memory destabilization and updating in the amygdala and hippocampus [16, 27, 28]. In brief, ubiquitin is activated by the ubiquitin E1 ligase and then transferred to ubiquitin conjugases E2, and the complex interacts with a ubiquitin ligase E3, finally conjugated to lysine residues within the substrate, or the N-terminal amino group. The complex then recognizes the targeted protein. The ubiquitinated targeted protein is recognized by the 26s proteasome or lysosome leading to substrate degradation [29, 30]. Thus, the activation of ubiquitylation and/or proteasome activity enhances the activity of the UPS. The retrieval of an aversive memory has been shown to activate the UPS through activating the proteasome activity in the amygdala [16]. In addition, CaMKII regulates the increases of both Rpt6-S120 phosphorylation and proteasome activity during memory reconsolidation [21].

In our previous studies, we have shown that CO_2_ inhalation during memory reconsolidation enhances the lability of the memory. The CO_2_ effects are through the activations of ASICs by which activating the intercellular Ca^2+^ signaling pathways, resulted in the turnover of AMPARs. The CO_2_ inhalation enhances memory retrieval within the reconsolidation window [6]. We thus asked if the CO_2_ inhalation and retrieval activate the same intracellular signaling pathways since both processes activated Ca^2+^-dependent signaling pathways. Interestingly, CO_2_ inhalation enhances the protein degradation without changing the proteasome activity through examining the proteasome activity and Rpt6-S120 phosphorylation in the amygdala following memory retrieval. However, previous studies suggested that both the protein ubiquitination and proteasome activity are increased in the amygdala following memory acquisition and retrieval [16]. We then further test if CO_2_ affects the process of ubiquitination. Indeed, when inhibiting ubiquitin E1 ligase with 50 µM Ziram, the exchange of the CI-AMPARs to CP-AMPARs has been decreased. This data suggest that CO_2_ inhalation enhances the reconsolidation through activating the ubiquitination process but not the proteasome activity. So, how does CO_2_ affect the UPS efficiency in protein degradation without increasing the proteasome activity? Classically, ubiquitination is a signal that directs proteins to the proteasome, where the ubiquitin is recycled and the protein is degraded [31]. Also, ubiquitination recasts the surface of the targeted proteins and changes the protein’s stability and activity [30], potentiates interactions with other proteins, and alters the subcellular localization [32]. Because ubiquitination might be particularly adapted to act as an efficient endocytic signal, the endocytosis of membrane AMPARs seems to be efficiently increased when enhancing the ubiquitin ligase E1.

Another interesting finding in this study is that the CO_2_ inhalation does not increase CaMKII phosphorylation after retrieval (Fig. 4). This is different from the mechanism of memory retrieval by which the CaMKII phosphorylation increases within the reconsolidation window [33]. Memory retrieval has been shown activated CaMKII phosphorylation to activate the AMPAR turnover. In addition, CaMKII regulates the UPS by activating the proteasome activity and the phosphorylation of the 19s regulatory subunit Rpt6, inducing protein degradation in the amygdala following memory retrieval [33]. Interestingly, evidence suggests that CaMKII only regulates the proteasome activities without directly affects the protein ubiquitination process [21]. We have previously shown that the effects of CO_2_ inhalation on memory retrieval are ASIC-dependent, which induces Ca^2+^ increase with the application of CO_2_. Of course, we cannot conclude whether the increase of intracellular Ca^2+^ was directly through ASIC1a or other Ca^2+^ channels. Because ASICs most likely form heteromeric sodium-selective channels and the calcium-permeable homomeric ASIC1a are limited in the brain. We used a powerful high throughput Kinome assay for kinase activity profiling in mice subjected to behavior treatment and found several Ca^2+^-dependent kinases with increased phosphorylation activity in response to CO_2_ inhalation in retrieval, suggesting potential signal molecules mediating acidification-induced signaling pathways in reconsolidation. Our studies in progress should further provide evidence elucidating how these kinases regulate UPS in such molecular events.

In all, our data demonstrated the cellular signaling pathway by which CO_2_ regulates the reconsolidation of aversive memories. Through activating ASICs, CO_2_ inhalation activates the ubiquitin mediated GluR2 degradation after memory retrieval without interfering with the proteasome activity in the amygdala neurons, different from the mechanism by which retrieval increases proteasome activity mediated by CaMKII (Fig. 6). Identifying the mechanism might have a substantial impact on our understanding of the mechanisms that underlie memory modification. This work is expected to lay the foundation for the development of novel therapies for mental illnesses such as post-traumatic stress disorder and anxiety.

## Data Availability

All the data supporting the conclusions of this article are included within the manuscript. Original data files and any supporting materials are available upon request, from the corresponding authors.

## Abbreviations

CO_2_: Carbon dioxide
CS: Conditioned stimulus
US: Unconditioned stimulus
ASICs: Acid-sensing ion channels
AMPAR: α-Amino-3-hydroxy-5-methyl-4-isoxazolepropionic acid receptor
CI-AMPAR: Ca^2+^ impermeabile-AMPAR
CP-AMPARs: Ca^2+^ permeable-AMPAR
GluR2: Ionotropic glutamate receptor 2
EPSC: Excitatory postsynaptic current
Av. C: Aversive conditioning
Ret: Retrieval
D-APV: D-2-amino-5-phosphonovalerate
NASPM: 1-naphthylacetyl spermine
PcTX-1: Psalmotoxin-1
β-Lac: Clasto-Lactacystin β-lactone
CaMKII: Ca^2+^/calmodulin-dependent protein kinase II
BSA: Bovine serum albumin
ATP: Adenosine triphosphate
KRSA: Kinome Random Sampling Analyzer
LFC: log2 fold change
IP: Immunoprecipitation
